# CAR-Based Therapy for Autoimmune Diseases: A Novel Powerful Option

**DOI:** 10.3390/cells12111534

**Published:** 2023-06-02

**Authors:** Györgyi Műzes, Ferenc Sipos

**Affiliations:** Immunology Division, Department of Internal Medicine and Hematology, Semmelweis University, 1088 Budapest, Hungary; dr.siposf@gmail.com

**Keywords:** chimeric antigen receptor, CAR T, CAR Treg, autoimmune, immune-mediated, CAR-based therapy

## Abstract

The pervasive application of chimeric antigen receptor (CAR)-based cellular therapies in the treatment of oncological diseases has long been recognized. However, CAR T cells can target and eliminate autoreactive cells in autoimmune and immune-mediated diseases. By doing so, they can contribute to an effective and relatively long-lasting remission. In turn, CAR Treg interventions may have a highly effective and durable immunomodulatory effect via a direct or bystander effect, which may have a positive impact on the course and prognosis of autoimmune diseases. CAR-based cellular techniques have a complex theoretical foundation and are difficult to implement in practice, but they have a remarkable capacity to suppress the destructive functions of the immune system. This article provides an overview of the numerous CAR-based therapeutic options developed for the treatment of immune-mediated and autoimmune diseases. We believe that well-designed, rigorously tested cellular therapies could provide a promising new personalized treatment strategy for a significant number of patients with immune-mediated disorders.

## 1. Introduction

In light of their prolonged nature, costly medical care, and expanding prevalence among individuals around the globe, autoimmune conditions pose a significant clinical problem. Depending on the discrepancy between effector and regulator immune responses, autoimmune reactions commonly undergo phases of remission and aggravation. The imperfect regulation and/or removal of autoreactive T and B lymphocytes, which ultimately leads to a breakdown of immune tolerance, is the primary cause of autoimmune diseases. Experimental models and human studies are shedding light on the genetic, environmental, and epigenetic factors that trigger autoimmunity. Utilizing this knowledge is a cornerstone for the discovery of novel therapies for restoring the equilibrium of aberrant immune functions.

Although autoimmune disorders are typically classified as T or B cell-mediated, both T and B cells contribute to the pathophysiology of many autoimmune diseases. T cells develop from lymphoid progenitors and are able to migrate from the bone marrow to the thymus. In this phase of T cell development, TCR-mediated selection and maturation into naïve T lymphocytes occur [1]. Lymphopoiesis begins with CD2-, CD5-, CD7-, and CD3-expressing progenitor cells capable of entering the thymus cortex. Rearrangements of α, β, γ, and δ chains lead to the formation of TCRs, resulting in γδT and αβT cells. At this point, NK T cells evolve from CD3+ precursor T cells by displaying a specific α chain that interacts with glycolipid-CD1d via a β chain [1,2]. Throughout the spectrum of T cell features, different subpopulations can be distinguished, including CD4+ αβ Th cells (i.e., Th1, Th2, Th9, Th17, and Th22), CD8+ αβ Tc cells (i.e., Tc1, Tc2, Tc9, and Tc17), CD8+ Treg cells, and their additional memory types (i.e., stem cell-, central-, and effector memory cells) [1].

B cells take responsibility for the short- and long-term production of humoral antibody responses, constituting a vital component of the immune system. Antigen presentation, regulation of T cell differentiation and survival, and the generation of regulatory and pro-inflammatory cytokines are additional antibody-independent functions performed by B cells [3,4]. The canonical B cell populations in the human peripheral blood can be classified according to the core markers CD19, IgM, IgD, CD27, CD24, and CD21. Peripheral B cell populations consist of transitional B cells (i.e., T1, T2, and T3 B cells), naïve B cells (i.e., resting, activated, and anergic), memory B cells (i.e., unswitched, pre-switched, switched resting, switched activated, and atypical tissue-based), double negative B cells, antibody-secreting cells (i.e., early plasmablasts, plasmablasts, and naïve, memory, resting, and mature plasma cells), Breg cells, PCreg cells, and natural antibody-producing B1 cells [4].

In autoimmune conditions, the main goal of targeting B cells would be to eliminate autoreactive effector B cells and boost autoantigen-driven Bregs while keeping the immune system under surveillance. It is difficult to carry out such a strategy, particularly because antigen-specific targeting is challenging and the relative importance of B cells to the pathophysiology of autoimmune conditions varies significantly between diseases. Current therapies for autoimmune diseases aim to suppress the immune system in part through the use of targeted therapies like small molecules (e.g., JAK inhibitors, TYK2 selective inhibitor, and BTK inhibitors) that inhibit immune cell activation or proliferation and in part by employing biological drugs (e.g., monoclonal antibodies) that target immune cells or their downstream effectors [5,6,7,8,9]. Nowadays, among the biologics the anti-CD20 rituximab is used frequently against B cell-mediated disorders [10]. To maintain remission, however, frequent rituximab infusions are required, leading to persistent B cell depletion and, subsequently, chronic immunological suppression resulting in infections and secondary tumor development [11].

### Adoptive Cell Transfer Immunotherapy Approaches

A promising and rapidly developing type of cell-based immunotherapy is adoptive cell transfer (ACT), an in vitro procedure that involves the cultivation of autologous extracted T cells for subsequent transfusion [12]. Various ACT techniques are being developed, including tumor-infiltrating lymphocyte (TIL) therapy, T cell receptor engineered T (TCR T) cell therapy, and chimeric antigen receptor T (CAR T) cell therapy.

Initially developed ACT was based on the isolation of tumor-specific TILs for ex vivo expansion and reinfusion into the patient [13]. This approach could only be feasible for resectable tumors from which sufficient T cells could be isolated and expanded [14].

In the so-called TCR-engineered cellular therapy, harvested T cells are infected with a retrovirus containing a copy of the TCR gene, which is specialized in recognizing tumor antigens. The retrovirus integrates the TCR gene copy into the T cell genome. The cells are then induced to divide and/or stimulated and eventually released back into the host, where they develop an immune response against the tumor cells [15].

**Figure 1:**
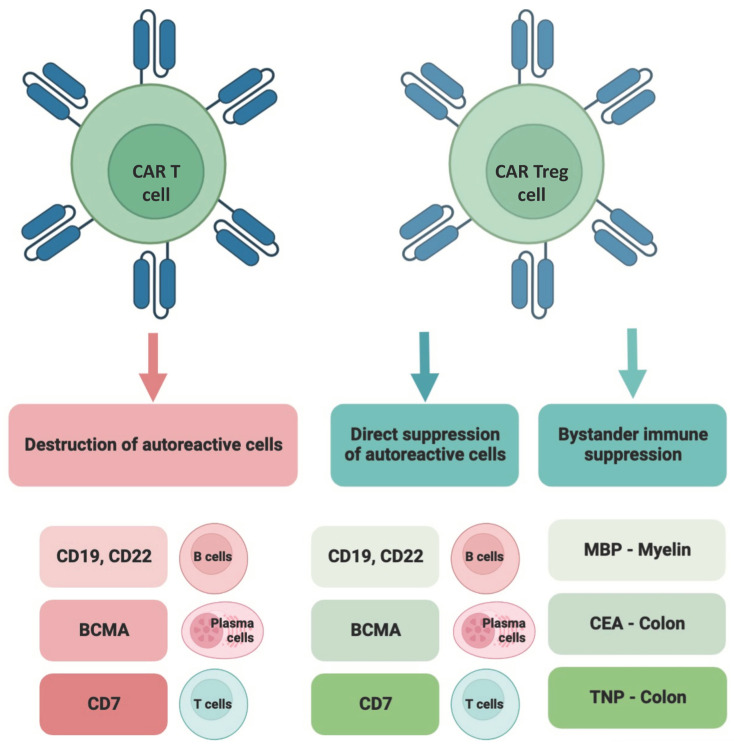
The comparison of CAR T and CAR Treg cell-based therapeutic strategies for immune-mediated and autoimmune diseases. CAR T cells can be used to destroy self-reactive cells. CAR Tregs, on one side, can suppress self-reactive cells. Regarding the destruction or suppression of autoreactive cells, CD19, CD22, BCMA, and CD7 belong to the most studied target molecules. CAR Tregs, however, can display their immunomodulating effects through bystander immune suppression. Taking this into account, MBP, CEA, and TNP are well-studied molecules. The figure was partly created by using www.biorender.com, accessed on 11 April 2023.

CAR T cells are T cell immunotherapies that have been genetically modified to express a synthetic receptor [16,17]. CARs regulate the lysis of antigen-expressing target cells and the development of CAR T cells into long-lived memory CAR T cells upon attaching to antigen-expressing target cells. CAR Treg therapy and chimeric autoantibody receptor T cell (CAAR T) therapy, which depletes antigen-specific B cells, are novel therapeutic variations of CAR T cells for autoimmune disorders [15,18] (Figure 1).

The development of CAR natural killer (NK) cell therapies was motivated by the adverse effects of CAR T cell therapies, such as GvHD, CRS, on-target/off-tumor effect, and neurotoxicity. This is due to the specific characteristics of NK cells, such as the restriction on HLA-matching and the absence of CRS, neurotoxicity, and GvHD [19]. Currently, the use of allogenic NK cells as a CAR platform therapy offers new doors only in the field of oncology [19].

In this review article, we provide a brief overview of the many CAR-based therapeutic options that have been created for the treatment of immune-mediated and autoimmune diseases. We believe that well-designed cellular therapies, tested under rigorous conditions, could provide a promising new personalized treatment strategy for a large number of patients with immune-meditated disorders.

## 2. Structural Design and T Cell Engineering

### 2.1. T Cells as the Basis of Engineering

T cells are adaptive immune cells that are essential for the elimination of infected and cancerous cells. T cells require initial stimulation through the interaction of a specific antigenic peptide with the TCR and MHC in order to become completely activated. Yet, T cells also require secondary co-stimulation from co-receptors and cytokine signaling for complete activation and differentiation. Several techniques are employed by cancer cells to circumvent or disrupt T cell function (e.g., lowering MHC presentation, anti-inflammatory cytokine release leading to T cell exhaustion, and immune cell suppression by inhibitory co-receptor signaling) [20,21]. By combining antitumor selectivity and T cell cytotoxicity, CAR T cells sidestep many of the limitations of cancer treatment.

Tregs are a subpopulation of CD4 T cells that regulate immunological tolerance. Tregs suppress the proinflammatory activity of CD4+ and CD8+ T cells, NK cells, and APCs. The powerful suppressive and metabolic regulatory activities of Tregs facilitate tissue healing [22,23,24]. Immune dysregulation and autoimmune disorders are triggered by disruption of Tregs [25]. Sustained high expression of the transcription factor Foxp3 in Tregs is required for suppressive action [25]. There are two primary categories of Foxp3+ Tregs: nTregs, which are professional cells that arise in the thymus, and pTregs, which differentiate from naïve CD4+ T cells in the presence of TGFβ [26]. Mutations in the Foxp3 gene in mice and humans result in Treg malfunction and severe autoimmunity (i.e., IPEX syndrome in humans) [27,28].

Cross-linking of CTLA-4, expressed by Tregs in the presence of TCR signaling increase the production of Foxp3+ T cells [29]. Moreover, CTLA-4 activation influences CD28 cross-linking, hence enhancing Foxp3 expression [29]. In tumors, CTLA-4 inhibition has anticancer effects, but it also increases autoimmunity [30]. The production of TGFβ, IL10, and IL35 by human Tregs influences surrounding immune cells [31]. Treg cells have become an appealing therapeutic option for the treatment of autoimmune diseases and for modifying or avoiding transplant rejection and GvHD due to their proved immunomodulatory capabilities.

In the past few years, there have been several Phase I clinical trials that looked at the immunotherapeutic potential, benefits, and risks of Treg-based ACT treatment. In several immune-mediated or autoimmune disorders, autologous ex vivo expansion of polyclonal Tregs has been explored [32,33,34,35,36]. Treg cells’ functional activity, stability, persistence, and antigen specificity can be improved through genome editing utilizing cutting-edge technology. In mouse models, antigen-specific or redirected Treg cells perform better than traditional polyclonal Treg cells, as demonstrated by preclinical investigations [37,38,39,40]. This is due to the fact that redirected CAR or TCR Treg cells are primarily localized at the region of target antigen expression and therefore pose a lower risk of systemic immunosuppressive effects. Also, the new techniques make it possible to produce Tregs from naïve CD4+ T cells through specific alterations, such as the activation of Foxp3 expression [40,41].

### 2.2. CAR T Cell Manufacturing

The CAR T cell manufacturing procedure consists of five stages [42]. First is the extraction and stimulation of T cells. CAR structure production and transmission is the second step, followed by CAR T cell proliferation in vitro. The fourth phase is the evaluation of the characteristics and functions of CAR T cells. The final step is cryogenic preservation and storage of CAR T cells until delivery to the patient [42].

**Figure 2:**
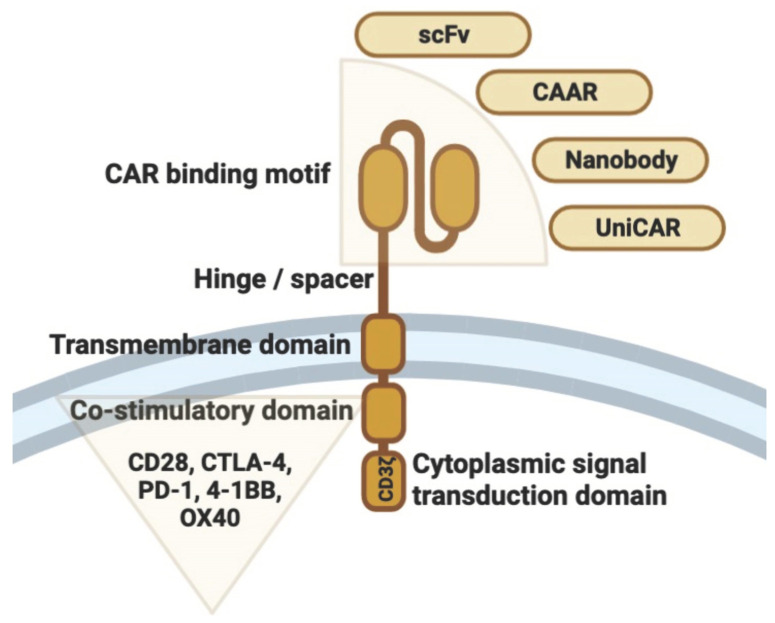
Schematic representation of the structure of the chimeric antigen receptor. The extracellular CAR-binding domain (e.g., scFv, CAAR, nanobody, or uniCAR) is linked to the transmembrane domain via a hinge region. This is linked intracellularly to a co-stimulatory domain (e.g., CD28, CTLA-4, PD-1, 4-1BB, or OX40) and a cytoplasmic signal transduction domain (i.e., ITAM: immunoreceptor tyrosine-based activation motif, e.g., CD3ζ or FcRγ). The figure was partly created by using www.biorender.com, accessed on 11 April 2023.

During CAR structure construction T cells are genetically modified to express a synthetic receptor with four key domains: (1) an antigen-recognition domain, such as an anti-CD19 single-chain variable fragment antibody; (2) an extracellular hinge or spacer domain; (3) a transmembrane domain; and (4) an internal domain composed of a co-stimulatory domain and a TCR cytoplasmic signal transduction domain [16,17] (Figure 2).

According to their source, CAR T cells can be classified as autologous (autoCAR T) and allogenic (alloCAR T). In the case of autoCAR T cells, T cells originate from the patients, so immunological rejection is absent [43]. However, CAR T exhaustion and consequent therapeutic insufficiency are quite common phenomena [44]. In addition, the whole procedure is highly time-consuming for some seriously ailing patients [42]. Conversely, alloCAR T cells, which originate from donors, can be harvested, processed, and administered to patients without delay. AlloCAR T cells have increased cytotoxic effects, and the entire procedure may be less expensive on average [42]. The main disadvantages of alloCAR T cells are GvHD and HvGD [45].

### 2.3. Generations of CARs

CAR T cells were initially created more than 40 years ago. In 1982, the first CAR prototype was developed. They had a TNP-specific scFv-binding domain connected to the CD3ζ or FcRγ signaling domains for T cell activation [46]. While scFv is the most prevalent extracellular domain, certain CAR designs also include a high-affinity TCR for the identification of intracellular TAAs or a nanobody domain derived from camelid antibodies. In the first generation of CAR T cells, there was no extra co-stimulatory domain, which is required for complete T cell activation. However, these CAR constructs were functional because T cells were able to compensate for the absence of the coactivating (2nd) signal via the natural CD28-B7.1/B7.2 connection between the T cell and the target cell [46,47].

In second-generation CAR constructions, the signal transduction domain has been fused with a coactivating domain [48,49]. Essentially, three distinct types of CAR T cells have been created. In one, the coactivating domain was CD28; in another, it was 4-1BB; and in the third, it was OX-40. In preclinical research, it was discovered that effector T cells expressing CARs carrying CD28 had a greater proliferation rate and produced more IL2, IFNγ, and TNFα than CAR T cells expressing 4-1BB [50,51,52]. It has also been demonstrated that the CD28ζ format has a beneficial effect on Tregs in the TME by eliciting increased IL2 production [53].

This raised the question of whether the incorporation of an extra costimulatory domain could improve the efficacy of CAR T cells, hence preventing TME-mediated T cell fatigue. This resulted in the production of CARs of the third generation. They consist of a scFv, a CD3ζ domain, and two costimulatory domains. According to the results of initial clinical trials, they have not yet demonstrated superior anticancer activity compared to CARs of the second generation [54].

Although second-generation CAR T cell-based immunotherapy has demonstrated promise, the anticancer effect has not been as prolonged as anticipated. To enhance their effectiveness against solid tumors, CAR T cells were genetically engineered. By activating CAR signaling, the aim was to achieve transgenic cytokine production in the targeted tumor tissue [55]. This resulted in the production of CAR T cells of the fourth generation, termed the TRUCK strategy. Hence, these cells combine the direct antitumor action of the CAR T cell with the TME-modulating capacity of a proinflammatory cytokine (e.g., IL12, IL7, IL15, or IL18). Activation of the CAR promotes phosphorylation of the NFAT, nuclear translocation, and stimulation of the NFAT-responsive/IL2 minimum promoter that controls transgene expression [55]. TRUCK cells may also be of interest in the therapy of autoimmune diseases, as the proinflammatory cytokine gene can be replaced by an anti-inflammatory cytokine gene (e.g., IL10, or TGFβ). Interestingly, in Tregs, activated NFAT1 forms a triple complex with Foxp3 at the IL2 promoter, which replaces AP-1 (Jun/Fos) in the AP-1 complex present in NFAT in effector T cells [56,57]. Thus, Foxp3 converts the transcriptionally activating NFAT/AP-1 complex in effector T cells into a repressive NFAT/Foxp3 complex in Tregs [57]. Also representing the fourth generation of cell therapies in addition to TRUCK CARs are “Armored CAR”, “Inducible CAR”, and “On/Off-Controllable VIPER CAR” therapies [19].

**Figure 3:**
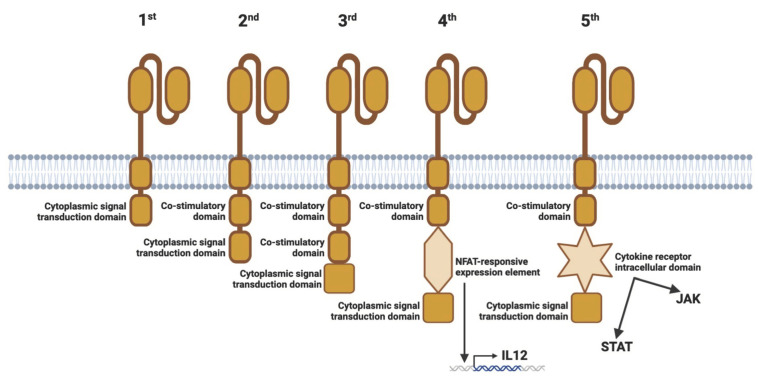
Generations of chimeric antigen receptors. The first-generation CARs have only one cytoplasmic signal transduction domain (e.g., CD3ζ). The second-generation CARs contain an intracellular co-stimulation domain (e.g., 4-1BB or CD28). The third-generation CARs have two co-stimulatory domains (e.g., CD28 and 4-1BB). The fourth-generation CARs additionally contain a NFAT-responsive expression element for an inducible transgenic product (e.g., an IL12-inducer element, leading to IL12 gene transcription). The fifth-generation CARs have an additional set of intracellular domains of cytokine receptors (e.g., IL2Rβ chain fragment, resulting in JAK/STAT activation). The figure was partly created by using www.biorender.com, accessed on 27 May 2023.

Fifth-generation CARs are also derived from second-generation cells, which have intracellular cytokine receptor domains. A CD19-specific CAR construct was constructed with a STAT3-binding tyrosine-X-X-glutamine motif integrated into the CD28-CD3ζ activation region [58]. In this type of CAR, a JAK-STAT signaling domain mediates effector function to stimulate persistence and memory, and generates the same stimulation as regular cytokine signaling. Inducing cytokine synthesis and stimulation via the JAK/STAT pathway elicits the same response from the cell as IL21 stimulation [58] (Figure 3).

It is necessary to mention that due to the limitations and side effects of CAR T cell therapy, such as GvHD, neurotoxicity, and cytokine release syndrome, researchers are investigating safer alternative cells, such as NK cells. The antitumor activity of CAR NK treatments has been demonstrated by in vitro and animal models [19]. Concurrently, the engineering of CAR constructs is fast evolving to produce “intelligent” tools to support and enhance existing CAR therapies. Several clinical trials utilizing NK cells and NK-like cell lines generated from blood or iPSCs are now active [19].

## 3. CAR-Based Therapy for Autoimmune and Immune-Mediated Diseases

In the following sections, we describe animal and human studies on the applicability and efficacy of CAR-based therapy for autoimmune and immune-mediated disorders, as well as ongoing or planned CAR clinical trials.

### 3.1. CAR T and CAR Treg Therapies and Clinical Trials in Systemic Lupus Erythematosus

SLE—an idiopathic, multifactorial, chronic autoimmune disease—is primarily characterized by immune dysregulation, antibodies to nuclear and cytoplasmic antigens, systemic inflammation with a broad spectrum of clinical manifestations, and a relapsing-remitting course [59].

Although CAR T has been used successfully in animal models of SLE, CAR Treg treatments are still uncommon, but both therapies show great promise [60]. Once CD8+ T cells targeting CD19+ B cells transduced with A-MLV retrovirus were transfused into MRL-lpr mice, CAR T cells decreased CD19 gene expression in the spleen, alleviated SLE symptoms, and increased the animals’ survival. Furthermore, CD19-targeted CAR T plasmids were transduced into splenic CD8+ T cells in NZBxNZW F1 mice and then infused into mice with SLE symptoms. The treatment induced CD19+ B-cell hypoplasia, demonstrating the therapy’s effectiveness [61]. CD19-targeted CAR T cell treatment with CD28 or 4-1BB co-stimulatory molecule CAR was also compared to anti-CD19 1D3 monoclonal antibody therapy in MRL-lpr mice. Anti-CD19 CAR T ACT resulted in a more prolonged reduction of B cells in mice than antibody treatment; moreover, the improved therapeutic effectiveness of CAR T cells with 4-1BB was demonstrated. It was also discovered that mice pretreated with mild doses of total body irradiation survived substantially longer [62]. Recently, a 20-year-old female patient with severe (SELENA score: 16), therapy-refractory SLE and type III/A lupus nephritis was treated with anti-CD19 CAR T therapy. After lymphodepletion (also known as conditioning treatment) with fludarabine to prevent immune rejection and to increase CAR T cell expansion and persistence, 1.1 × 10^6^ anti-CD19 CAR T cells were supplied per kilogram of body weight (a CD4+ to CD8+ T cell ratio of 3:1). Five weeks after CAR T cell infusion, the patient’s health improved, the dsDNA autoantibody titer and C3 and C4 complement levels normalized, and proteinuria with nephrotic grade was virtually eradicated. The score for SELENA was 0. In addition, no substantial adverse effects were noted [63]. Afterward, the same group treated an additional four SLE patients with refractory disease. All patients achieved the LLDAS and were able to discontinue all SLE-specific medications, including glucocorticoids, based on preliminary safety and efficacy data. No SLE flare has occurred to date; however, long-term follow-up data are required [64].

In a recent study [65], five patients (four women and one man) with long-standing, high disease activity and therapy-refractory SLE were enrolled in an anti-CD19 CAR T cell program as a last resort. Autologous T cells from the SLE patients were transduced with a lentiviral anti-CD19 CAR vector, expanded, and then returned to the patients at a dose of 1 × 10^6^ CAR T cells/kg body weight after lymphodepletion with fludarabine and cyclophosphamide. SLE remission according to DORIS criteria occurred in all five patients after 3 months, and the median range of the SLEDAI score was 0 after 3 months. Drug-free remission was maintained beyond 8 months. The B cells that reappeared after a median of 110 days were naïve and were not-class-switched B cells. The CAR T cell treatment was well tolerated, with only mild CRS observed.

**Figure 4:**
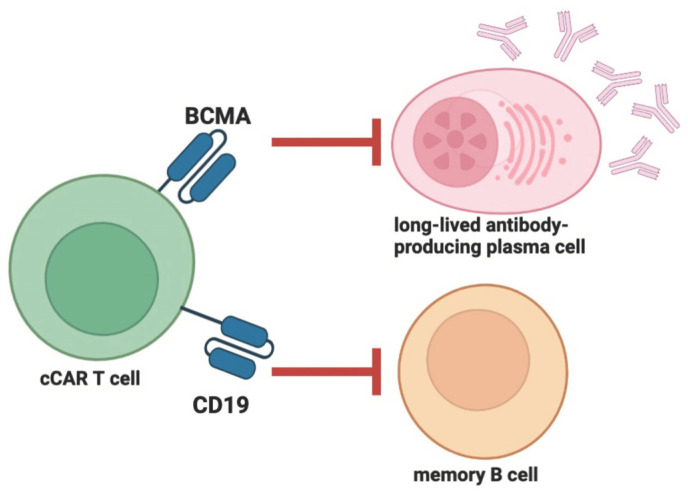
The compound CAR construction is a two-unit CAR consisting of an entire BCMA-CAR linked to a full CD19-CAR, allowing for the autonomous expression of each of the CAR receptors on the T cell surface. This enables the CAR T cell to target long-lived antibody-producing cells (i.e., CD19+ memory B cells and BCMA+ plasma cells). Red arrows indicate inhibition. The figure was partly created by using www.biorender.com, accessed on 28 May 2023.

In another study, a 41-year-old female patient with stage IV DLBCL and a 20-year history of SLE was treated with compound CAR T (cCAR T) co-expressing anti-BCMA and anti-CD19 bearing the CD137 co-stimulation domain [66]. Prior to the cCAR T infusion, the patient was preconditioned with fludarabine and cyclophosphamide. SLE remained in remission without additional treatment for 20 weeks after treatment, the ANA titer remained negative for 37 weeks, and B cells began to repopulate approximately 28 weeks after treatment (Figure 4).

According to www.clinicaltrials.com accessed on 11 April 2023, there are currently four clinical trials registered to evaluate CAR T therapy in SLE. The first research study (NCT03030976) is being carried out to assess the safety and efficacy of anti-CD19 CAR T cells in the treatment of SLE patients. Using CD19 as the target and 4-1BB as the co-stimulator, the researchers built a second-generation CAR and adjusted its spatial structure with an appropriate hinge and transmembrane domain sequences. The second study (NCT05030779) also focuses on the B cell-killing effect of cCAR T cells. It aims to investigate the safety and efficacy of CD19/BCMA cCAR T cells in the treatment of refractory SLE. In the third study (NCT05474885) the number and incidence of adverse events after BCMA/CD19 cCAR T cell infusion will be monitored. The research group will evaluate all possible adverse reactions, including the number, incidence, and severity of symptoms such as CRS and neurotoxicity, within 3 months after CAR infusion. The fourth study (NCT05765006) is a Phase I, open-label, single-arm, multicenter trial to assess the safety, tolerability, pharmacokinetics, and pharmacodynamics of anti-CD19 CAR T therapy (Relma-cel) in patients with moderate-to-severe active SLE. The study will use 4 dose levels to assess dose escalation, safety, and tolerability.

Regarding CAR Treg cells, the immunological milieu of an SLE patient provides numerous difficulties [67]. High levels of IL6 and IFNα secreted by DCs impair Treg activity, while IL21 secreted by CD4+ T cells is equally deleterious to Treg survival and function [68]. Strong proinflammatory conditions can transform Treg cells into IL17 (or other inflammatory cytokine)-producing cells.

Although numerous autoantibodies may be expressed in SLE, CAR Treg may be effective in restoring Treg numbers and related inhibitory activities. Infusion of autologous Tregs can activate Tregs in inflamed skin, thereby inhibiting the IFNγ pathway and CD4+ effector cell invasion. It has been demonstrated that low-dose IL2 treatment in sixty individuals with active SLE is successful and restores tolerance [69]. Based on these findings, CAR Treg treatment can restore immunological tolerance and reduce inflammation in skin and renal tissues.

### 3.2. CAR T and CAAR NK Therapies and Clinical Trials in Sjögren’s Syndrome

SS is a systemic autoimmune disorder characterized by persistent inflammation of the exocrine (mainly salivary and lacrimal) glands. It is often accompanied by systemic symptoms. B cell hyperactivity is indicated by the presence of different autoantibodies, such as RF and anti-SSA/SSB antibodies, as well as hypergammaglobulinemia [70]. Five to ten percent of patients with SS develop malignant B cell lymphoma, most commonly of the mucosa-associated lymphoid tissue subtype and most often affecting the major salivary glands [71]. This accurately depicts the potential applicability of CAR T cells in SS.

CAR T therapy has not yet been tested in an animal model of SS. However, there is a single-arm, open-label clinical trial (NCT05085431) of CD19/BCMA cCAR T treatment to assess its safety and efficacy. The primary endpoint of the clinical trial is dose-limiting toxicity and the emergence of treatment-related adverse events.

In addition, a La/SSB-reactive B cell-targeting CAAR NK strategy has been reported [72]. Approximately 25–40% of patients with SS show anti-La/SSB autoantibodies [73]. An NK cell line (NK92MI) has been engineered to express CAAR containing the immunodominant domain of the La/SSB protein [72]. Although in vivo data have not been reported, La-CAAR NK92MI cells showed selective cytotoxicity against anti-La BCR+ target cell lines and a partial reduction in B cell frequency by flow cytometry compared to healthy controls after whole blood samples obtained from anti-La seropositive patients were co-cultured with La-CAAR NK92MI cells.

### 3.3. Compound CAR T Therapy and Clinical Trials in ANCA-Associated Vasculitis and Autoimmune Hemolytic Anemia

ANCA-associated small vessel vasculitides are the autoimmune inflammatory diseases of the vascular wall in which randomized controlled trials of rituximab and cyclophosphamide have shown the superiority of rituximab in inducing 6 and 24 months of remission of relapsed disease [74,75]. This suggests that repeated rituximab infusions may be needed to maintain disease control and that CAR T therapy may provide a more durable therapeutic option.

AIHA is a cell-specific autoimmune disease characterized by severe anemia due to autoantibodies against red blood cells, which are IgG isotypes in warm AIHA and IgM isotypes in cold AIHA. Treatment with rituximab has beneficial effects in both types, although randomized controlled trials with rituximab have not yet been performed in AIHA [76,77].

The reason for mentioning the two autoimmune diseases together is that there is only one clinical trial involving patients with vasculitis or AIHA (in addition to other diseases such as POEMS syndrome or amyloidosis). In this study (NCT05263817) the safety and effectiveness of CD19/BCMA cCAR T treatment of refractory diseases are assessed. However, based on the results of CAR-based treatment of SLE, it is hypothesized that further promising therapeutic results could be achieved in ANCA-associated vasculitis and AIHA using CAR T cell therapies, but further clinical trials are needed.

### 3.4. CAR T and CAR Treg Therapies in Rheumatoid Arthritis

RA is a chronic systemic inflammatory disease marked by persistent symmetric polyarthritis (synovitis) affecting primarily small joints. The synovial membrane is the target of an autoimmune reaction. Significant extra-articular involvement may also occur in organs such as the skin, heart, lungs, and eyes [78]. ACPA have been thoroughly studied in RA and may have a pathogenic function. In mice, ACPA against citrullinated vimentin may increase osteoclast genesis and bone resorption, indicating a pathogenic role for B cells [79,80]. Rituximab has been shown to be effective in patients with active RA, particularly in those with high ACPA levels [81]. Consequently, it is logically hypothesized that the formation of CAAR T cells expressing citrullinated antigens would enable the elimination of anti-citrulline B cells.

In an in vitro study, it was demonstrated that anti-FITC CAR T cells could be precisely redirected and kill hybridoma cells created by immunization with antigenic peptides and autoreactive B cell subsets from RA patients by recognizing appropriate FITC-labeled citrullinated peptide epitopes. Moreover, the cytotoxicity of CAR T cells was dose-dependent and reliant on the presence of peptides [82,83].

Developing particular CAR Tregs that induce tolerance in the synovium of affected joints also appears to be a potential option. CV is a particular antigen discovered only in the extracellular matrix of the inflamed synovial tissue of RA patients [84]. According to unpublished preliminary results, engineered CAR Tregs targeting CV may react with CV expressed in RA synovial fluid. However, further research is required to explore the effect of CAR Tregs in preclinical RA models [85].

Unfortunately, there are currently no ongoing clinical trials for CAR-based therapy for RA.

### 3.5. CAR T Therapies and Clinical Trials in Systemic Sclerosis

SSc is a progressive systemic connective tissue disease characterized by autoimmunity, vasculopathy, excessive extracellular matrix deposition and fibrosis, and consequent atrophy of the skin, subcutaneous tissue, muscles, and internal organs (e.g., digestive system, lungs, heart, kidneys, central nervous system) [86]. In SSc, immune cells, including T and B cells, and macrophages, display a variety of immunological abnormalities, as demonstrated by numerous recent investigations [87,88].

The European Scleroderma Study and Research Group published a clinical trial regarding the effect of rituximab on the fibrosis of the skin and lungs [89]. The study showed an improvement in skin fibrosis and a prevention of the progression of pulmonary fibrosis. In an open comparative study, the efficacy of rituximab and oral immunosuppressive therapies (i.e., azathioprine, methotrexate, and mycophenolate mofetil) was studied [90]. Clinical improvement in SSc-associated interstitial lung disease and skin fibrosis was also observed in the group treated with rituximab. In a double-blind, placebo-controlled trial with rituximab, 56 patients with SSc were studied (rituximab vs. placebo). Participants in the rituximab group experienced a significant decrease in the modified Rodnan Skin Score and a progression in the placebo group [91].

Studying the anti-fibrotic effects of CAR T cells found interesting preclinical results. In the mouse model of heart fibrosis, the activity of CAR T cells is directed against the protein that activates fibroblasts. In this model, CAR T CD8+ cells successfully destroyed the heart fibroblast that expressed the xenogeneic antigen [92]. Further examining this approach, during the in vivo ACT of anti-fibrotic CAR T cells, modified messenger RNA was injected into the cells in T cell-targeted lipid nanoparticles in order to increase safety [93].

Despite these promising results in preclinical models, clinical trials in patients SSc are still preliminary. The safety and efficacy of treatment with CD19/BCMA-CAR T were assessed in an open clinical trial (NCT05085444) for therapy-refractory SSc. The primary objective of this study is to investigate dose-restricting toxicity and the incidence of adverse reactions.

### 3.6. CAR T, CAAR T and CAR Treg Therapies and Clinical Trials in Immune-Mediated Neurological Disorders

MS is an inflammatory autoimmune demyelinating disease of the CNS in which a large number of patients develop significant neurologic disability, such as weakness, vision loss, and cognitive decline. The spectacular effectiveness of systemic anti-CD20 B cell-depleting monoclonal antibodies in treating relapsing-remitting MS demonstrated that B cells play an essential role in the pathogenesis of the disease [94,95].

Anti-CD19 CAR T cells were studied in an EAE animal model of MS [96]. In this scenario, mice spontaneously acquire EAE accompanied by meningeal B cell aggregates. It was discovered that anti-CD19 CAR T cell therapy decreased the size of B cell aggregates in the meninges but aggravated the clinical illness. This is comparable to what was reported when anti-CD20 B cell depletion caused paradoxical worsening in the same spontaneous EAE or in EAE generated by immunization with MOG peptide (p)35–55 [97,98]. Recent research examined anti-CD19 CAR T cells in a B cell-dependent model of EAE caused by immunization with the extracellular domain of rhMOG protein [99]. Without causing systemic toxicity, this work established the efficacy of anti-CD19 CAR T cells in treating a B cell-dependent model of EAE. Although anti-CD20 monoclonal antibody therapy decreased B cells in both the CNS and the periphery in this mouse model, it is known that anti-CD20 antibodies do not effectively reach the CNS in humans, whereas anti-CD19 CAR T cells do [100,101,102]. Using anti-CD19 CAR T cells, severe and durable B cell depletion has been achieved not only in the periphery but also in the central nervous system, demonstrating that anti-CD19 CAR T cells may hold promise for patients with particular autoimmune disorders [99].

Treg cell therapy has been efficient in the EAE mouse model of MS. In EAE animals, CAR Tregs directed against the antigen MOG exhibited suppressive capability in vitro and could efficiently target several brain areas via intranasal cell delivery. After a second assault with the MOG peptide, the animals exhibited decreased illness symptoms and brain inflammation and remained healthy, indicating that altered Tregs have persistent benefits [103].

Tregs, which express a TCR specific for the MBP, have been constructed as a result of previous developments in leukemia CAR therapy. These TCR-engineered specific Tregs inhibited the proliferation of MBP-reactive T effector cells and ameliorated EAE elicited by MOG [104]. Subsequently, this strategy was expanded by engineering human Tregs to express a functional scFv CAR against MBP or MOG. These scFv CAR-transduced Tregs maintained Foxp3 and Helios levels unique to Treg cells following prolonged in vitro expansion. In addition, these modified CAR Tregs were able to decrease autoimmune pathology in EAE, suggesting that they may be a viable therapy option for MS patients [105].

Conventional ex vivo CAR Treg training necessitates a vast amount of equipment and knowledge, making its implementation for patients with demyelinating disorders challenging. By translating the majority of the ex vivo processes to in vivo, a novel theoretical notion could make CAR Treg therapy viable. The lentivirus HIV, possessing tropism toward CD4+ T cells, is extensively modified and utilized as a vector to introduce particular genes, such as the CAR gene, into cells [106]. Changes include providing the envelope with desired proteins (i.e., pseudotyping), removing its pathogenicity, transmissibility, and replication strength, and equipping the envelope with the appropriate proteins [107]. By using pseudotyping, the lentiviral envelope can be filled with any protein, even designer proteins. The envelope of the lentiviral vector carrying the CAR gene is commonly pseudotyped with vesicular stomatitis virus G protein to ensure broad tropism and, hence, success in currently utilized CAR T cell therapy. Current CAR T cell generation utilizes the ex vivo presentation of lentiviral vectors to T cells; however, viral vectors can be delivered in vivo (e.g., adenoviral vector vaccines against COVID-19). Pseudotyping lentiviral vector envelopes with designed ligands of Treg-specific pairings such as CD25 and CTLA-4 can give Tregs their unique tropism, allowing them to be supplied in vivo as vectors for the CAR gene, leading to the production of host-produced CAR Tregs [108]. The following notion would make Treg therapy viable by shifting the synthesis of CAR Tregs in vivo, leaving only the mass manufacturing of Treg-specific CAR gene lentiviral vectors. According to www.clinicaltrials.gov accessed on 11 April 2023, there are currently no ongoing clinical trials in MS to test the effectiveness of CAR-based therapy.

MG is a chronic autoimmune neuromuscular disease caused by autoantibodies against the AChR, MuSK, or low-density LRP4 expressed in postsynaptic muscle cells [109]. About 80% of patients with MG show anti-AChR antibody positivity, and about 40% of the anti-AChR antibody-negative patients show anti-MuSK antibody positivity. The presence of anti-LRP4 autoantibodies can be detected among patients outside the previous groups [110]. Based on the localization of the affected muscles, ocular and generalized MG can be distinguished. Passive transmission of autoantibodies may be involved in the development of the disease, indicating a primary role for autoantibodies in the pathogenesis of the disease [111].

NMOSD is a rare, progressive demyelinating disease that affects the optic nerves, spinal cord, and less frequently the brain. NMOSD can induce a variety of symptoms, including visual loss, paralysis, persistent hiccups, nausea, and vomiting. Typically, symptoms improve after the initial episode. A blood test for aquaporin-4 IgG antibodies is highly specific for NMOSD and facilitates diagnosis, but some patients with NMOSD may not have detectable antibodies despite exhibiting the disease’s defining characteristics [112].

CIDP is an autoimmune disease that affects peripheral nerves and nerve roots and is characterized by a symmetrical loss of motor and sensory function [105]. Various autoantibodies (including NF155, CNTN1, CASPR1, NF140, and NF186) may be present in the disorder. CIDP is currently treated off-label with glucocorticoids, intravenous immunoglobulin, plasmapheresis, and rituximab [113,114].

IMNM is a subclass of idiopathic inflammatory myopathies, distinguished by elevated blood creatine kinase and necrotic muscle fibers, and associated with autoantibodies against SRP or HMGCR [115,116]. Patients with anti-SRP antibodies respond better to rituximab treatment than those with anti-HMGCR antibodies [116].

Although there have been no experimental model trials to test CAR-based treatments for MG, NMOSD, CIDP, and IMNM, there are three clinical trials investigating this new therapy. In a Phase IIb study (NCT04146051), the safety, tolerability and preliminary efficacy of a repeated dosing schedule of Descartes-08 CAR T cells are evaluated in patients with generalized MG. In a so-called basket trial (NCT04561557) BCMA-CAR T treatment of autoimmune inflammatory neurological diseases (i.e., MG, NMOSD, CIDP, and IMNM) are investigated. In this study, the safety, dose-limiting toxicity and efficacy of a novel CAR T cell therapy using CT103A cells are evaluated in patients with relapsed/refractory antibody-mediated idiopathic inflammatory diseases.

A Phase 1 trial (NCT05451212) is investigating the safety and toxicity of different dosing regimens of an investigational cell therapy called MuSK-CAAR T, which can be given to patients with active, anti-MUSK antibody-positive MG. The different dosing regimens of MuSK-CAAR T are being evaluated alone, in combination with cyclophosphamide and in combination with cyclophosphamide and fludarabine.

### 3.7. CAAR T Therapies and Clinical Trials in Pemphigus Vulgaris

PV is a severe, blistering autoimmune illness that affects the skin and mucous membranes. PV is mediated by Dsg-specific IgG autoantibodies. Autoantibodies bind to keratinocyte adhesion proteins Dsg1 and Dsg3 and impede keratinocyte adhesion, which results in lysis of the spinous layer and blister development. Dsg3 plays an essential role in PV [117].

Based on mice experiments and in vitro results, CAAR T cells made by fusing Dsg3 and CD137-CD3ζ were most effective when they expressed extracellular cadherin domains 1–4 from Dsg3 [118,119,120]. This CAAR T cell directly killed the memory B cells that express anti-Dsg3 sIg and eliminated indirectly the plasma cells that made short-lived, harmful anti-sIg-Dsg3 antibodies. Dsg3-CAAR T cells were not destroyed in the presence of pathogenic IgG, displayed mature specificity, and targeted only pathogenic B cells. In a mouse model, CAAR T cells drastically reduced the amount of anti-Dsg3 IgG-producing B cells without affecting the total number of pathogenic B cells [121]. This showed that CAAR T cells are able to find and kill the harmful anti-Dsg3 B cells in pulmonary vascular disease [109,112]. In mouse models, the T threshold dose of Dsg3-CAAR correlates, suggesting that a prudent fractional starting dose should be employed in future clinical trials [118].

Current standard clinical care for PV with corticosteroids, immunosuppressive medications, and rituximab requires a quite lengthy period to achieve remission, and relapse is common [122,123,124]. Several favorable aspects of CAAR T therapy have been identified, though. In CAAR T-cell experiments, no off-target adverse effects were observed. In addition, anti-Dsg3 B cells are unlikely to cause CRS since they represent a small proportion of total B cells in patients [125,126]. Furthermore, CAAR T cells are “living” agents capable of proliferating and persisting in vivo [118].

Up to a dose of 2.5 × 10^9^ CAAR T cells, preliminary clinical results from the first four cohorts of the DSG3-CAAR T research revealed no dose-limiting adverse effects [127]. At 28 days post-infusion, a dose-dependent rise in DSG3-CAAR T persistence approached the lower range of persistence values (observed in responders receiving anti-CD19 CAR T and lymphodepletion for B-cell leukemia) [128]. This implies that soluble antibodies do not cause CRS and do not destroy CAAR T cells. After DSG3-CAAR T infusion, transient improvements in clinical disease activity and antibody levels with a duration of two months were also seen [127].

**Figure 5:**
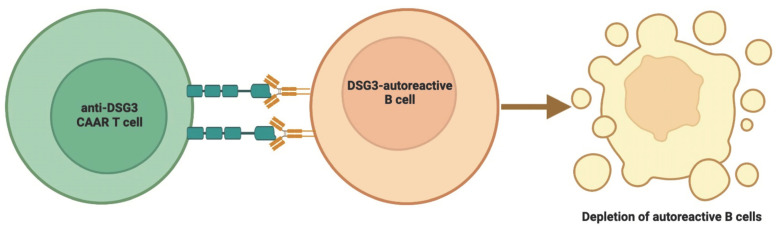
Chimeric autoantibody receptor T cell therapy depletes antigen-specific B cells. The figure was partly created by using www.biorender.com, accessed on 28 May 2023.

One Phase 1 study (NCT04422912) has been conducted to find the maximum tolerated dose and optimal fractionated infusion schedule of the DSG3-CAAR T therapy, that can be given to patients with mucosal-dominant PV who are inadequately managed by standard therapies (Figure 5).

### 3.8. CAR T and CAR Treg Therapies and Clinical Trials in Dermatomyositis, Adult-Onset Still’s Disease, and Inflammatory Bowel Disease

DM is an idiopathic inflammatory myopathy that affects children and adults and is characterized by unique cutaneous manifestations. This systemic disorder affects the skin and muscles most frequently, but it can also affect the joints, esophagus, lungs, and, less frequently, the heart. The endothelium of the endomysial blood vessels is believed to be the target of an immune response in DM. The pathophysiology of the cutaneous manifestation of DM is poorly known, but it has been hypothesized to be comparable to that of muscle involvement [129].

AOSD is an uncommon inflammatory disorder with an unknown etiology that primarily affects young individuals. It is distinguished by high spiking fevers, arthritis, and an ephemeral, nonpruritic, macular, salmon-colored rash on the trunk and extremities. Organomegaly, lymphadenopathy, serositis, and aseptic meningitis are also possible. Important laboratory results include leukocytosis with a preponderance of neutrophils, negative testing for rheumatoid factor and antinuclear antibodies, as well as elevated serum ferritin and decreased serum glycosylated ferritin [130].

IBD is an idiopathic immune-mediated disease resulting from a dysregulated immune response to the intestinal microbiota. In IBD, the intestinal homeostasis is chronically compromised, and an inflammatory immune response is constitutively active due to disruption of the intestinal epithelial barrier. The two most common forms of IBD are UC, which is restricted to the colonic mucosa, and CD, which can affect any segment of the gastrointestinal tract, is transmural, and involves “skip lesions.” There is a genetic propensity for IBD, and patients with this condition have an increased risk of developing cancer [131].

T cells play an important role in the pathogenesis of all three diseases [132,133,134].

An important area for CAR-based therapies is the potential to treat T cell-mediated autoimmune diseases. To avoid T cell fratricide, anti-CD5/CD7 CAR T therapies are based on the suppression of endogenous CD5 and/or CD7 expression (e.g., by using CRISPR/Cas9-based gene editing or intracellular retention of CD7 by a CD7-binding protein) [135,136]. A high rate and fairly durable remission were achieved in a clinical trial involving subjects with relapsed or refractory T cell acute lymphoblastic leukemia (NCT04689659) with CD7-CAR T, while CD7-negative T cell expansion was observed in the subjects. This suggests selective cytotoxicity and persistence of CD7-CAR T cells [136].

NKG2D-CAR T cells gain selectivity for tumor-expressed stress-induced ligands. However, these stress ligands are also transiently expressed by activated T cells, suggesting that NKG2D-based T cells may undergo self-killing prior to infusion into patients during cell production. To prevent target-driven fratricide and permit the generation of NKG2D-CAR T cells for therapeutic use, two independent techniques were evaluated [137]. The first involved incorporating a phosphoinositol-3-kinase inhibitor into the manufacturing process. A second technique included the use of antibodies to block NKG2D itself. Both procedures had an influence on T cell fratricide, but to varying degrees, with the antibody approach having the most impact on cell production. Accordingly, target-driven CAR T fratricide can be overcome by inhibiting the expression of NKG2D or the action of enzymes in general [137]. CAR T cells engineered using this technique are already being tested in cancer, but trials in autoimmune diseases are still to come.

CD7-negative T cells can also be found in psoriasis, RA, or adult-onset autoimmune enteropathy [138,139,140]. In the latter disease, a decrease in the CD8+CD7− T cell population is associated with clinical improvement [140].

Currently, one basket clinical trial (NCT05239702) is underway to evaluate the safety and efficacy of CD7-CAR T treatment in T cell-dependent autoimmune diseases (i.e., CD, UC, DM, and AOSD). The PREDICT trial (NCT03369353) will investigate the immunology of auto- and alloimmune gastrointestinal disorders, such as IBD, GvHD, and functional gastrointestinal disorders, as well as the immunological manifestations following CAR T and other cellular therapies.

Adoptive transfection of Tregs can prevent and treat autoimmune disorders caused by abnormal Treg cells in animal models [141]. Using a transgenic method, Tregs were made to express a CAR with a TNP-specific antibody-converting region fused to the extracellular and transmembrane domains of the co-stimulatory molecule CD28 and the intracellular domain of the stimulatory receptor chain Fc-γ (TNP-TPCAR Tregs) [142]. All T cells of transgenic mice expressing this receptor were resistant to TNBS-induced colitis. In the inflamed colons of non-transgenic animals receiving tiny numbers of TPCAR Tregs, the cells aggregated and became activated. Due to their antigen specificity, lack of MHC limitation, and independence from co-stimulatory signals, TNP-TPCAR Tregs could heal acute experimental colonic inflammation [143]. TNBS have also been utilized to inhibit colitis in different types of colitis models. Although TPCAR-containing Tregs did not prevent oxazolidone-induced colitis, oxazolidone-induced colitis was healed when a minimal quantity of TNBS was added, showing the bystander effect of TNBS [143].

TNP-TPCAR Tregs may show promise against normal human Tregs. However, the resistance of nTregs to viral vector transduction poses a challenge for the production and clinical application of TNP-TPCAR Tregs [144]. In addition, adoptive transfer of engineered Treg cells can be risky, as the in vivo inflammatory environment can trigger the conversion of Treg cells into antigen-specific pathogenic effector T cells. To address this problem, techniques such as CRISPR can be used for gene editing [145].

Transduction of specific CEA-SCA431 CAR signaling domains fused to CD28-CD3ζ into CD4+CD25+ Treg cells, yields CEA-CAR Tregs [146]. CEA-CAR Tregs are effective at attenuating T cell metastatic colorectal inflammation and inhibiting the development of AOM-DSS-induced colorectal cancer [143]. CEA-CAR Tregs can dwell and aggregate at locations expressing CEA, mostly in the inflammatory colon, with considerably lower amounts in the small intestine and other organs [146]. They are advantageous due to their high antigen specificity, lack of MHC limitation, specific proliferation, independence from co-stimulatory signals, precise targeting, and quick activity. Therefore, CEA-CAR Tregs have significant potential for treating colitis and preventing colitis-associated cancer. However, the longevity of CEA-CAR Tregs is limited (7–9 days) [147]. This could be the result of an immune response to possible epitopes on CAR or luciferase reporter proteins. The relatively short in vivo lifespan of CAR Tregs may be a consequence of activation-induced cell death [146]. Presently, CEA-CAR Tregs cannot be used clinically to treat colitis due to their limited lifespan, and clinical trials have not yet been published.

### 3.9. CAR T Therapy in Type 1 Diabetes Mellitus

T1D is a chronic autoimmune condition that destroys pancreatic β-cells that produce insulin [148]. Consequently, insulin deficiency ultimately results in hyperglycemia. Repetitive insulin injections suggest that insulin levels in affected people are not stable and that T1D patients require long-term treatment [148].

To date, T1D CAR treatment has been primarily focused on CAR-T therapy with limited use of CAR Treg. Chimeric MHC molecules augmented with TCR-signaling motifs serve as activation receptors and can steer gene-modified T cells against pathogenic CD8 T cells. In NOD mice, it was shown that CD8+ T cells may be reprogrammed to identify diabetogenic T cells by electroporation of mRNA expressing peptide/β2-microglobulin/CD3ζ. These CAR T cells can target autoreactive cytotoxic T cells in vivo to minimize insulitis and avoid autoimmune diabetes [149]. Similarly, in NOD mice, it was observed that monoclonal antibody-287-targeted CAR T cells retained their specificity and destroyed antigen-presenting cells in vitro. In vivo, they were able to delay the onset of T1D in a well-established preclinical model, although the protection faded with time [150]. In addition, it has been shown that functional CARs against insulin can be created and that the combination of CARs and Foxp3 can generate antigen-specific Tregs from naïve CD4+ effector T cells. The insulin-specific CAR Tregs exhibit the same phenotype and function as their natural counterparts. In NOD mice, insulin-specific CAR Tregs had a lengthy lifespan [151].

Although animal trials seem promising, there are no data on human investigations or ongoing clinical trials of T1D CAR-based therapy.

### 3.10. CAR Treg Therapy and Clinical Trials in Graft-Versus-Host Disease

GvHD is a multiorgan complication of ACT that is potentially fatal. It depends on the fact that the host seems non-self to the graft, allowing it to stimulate antigenically using a wide range of immunological processes. GvHD is categorized as acute and chronic [152]. Frequently, the chronic form mimics autoimmune conditions.

GVHD is one of the side effects of CAR-based treatments, however, a recent study has shown in vitro the suppression of IgG antibody production and differentiation of B cells by CD19-CAR Tregs. In the same study, infusion of CD19-CAR Treg in immunodeficient mice reconstituted with human peripheral blood mononuclear cells, suppressed their antibody production and reduced the risk of GvHD [153].

There is one clinical trial (NCT03369353) going on right now that is looking at the immunology of auto- and alloimmune gastrointestinal disorders, such as GvHD, and the immunobiological effects of CAR T and other cellular therapies. Table 1 illustrates the key features of CAR-based treatments for autoimmune diseases to date and the parameters of clinical trials.

## 4. Limitations of CAR-Based Therapies

The limitation of CAR-based treatments is partly due to technical and partly biological effects.

CAR T cells are difficult to generate and manufacture cost-effectively. The technique is laborious and costly, requiring a substantial amount of work and laboratory expertise. Additionally, CAR T cell therapy receives criticism for its high price [154,155]. However, these barriers to CAR T cell treatment might be overcome by advancing manufacturing technology and creating more sophisticated generations, such as uniCAR T cells, subsequent generations, and CAR NK cell therapy [156]. Further, emerging techniques for rapid manufacturing strategies, such as FasTCAR and T-Charge systems, may reduce the turnaround time and price of the therapy [157]. T-Charge is a newly discovered innovative approach for expanding CAR T cells. It is an infrastructure of the forthcoming generation that expands CAR T cells in vivo, thereby eliminating the need for a prolonged culture time ex vivo [158]. DNA nano-vectors, an emerging technology that allows for the rapid fabrication of CAR T cells on a therapeutic scale, have also been created [159]. DNA nano-vectors, which are non-viral and non-integrating miniature vector systems able to replicate extrachromosomally in the nucleus of dividing cells, improve the ability to create modified human T cells effectively. Therefore, it is a secure, effective, and reliable method for manufacturing modified T cells, which could reduce the cost of CAR T cell therapy. To make these treatment modalities more accessible, however, additional advancement in technology is required.

Allogenic CAR T cells may induce an autoimmune reaction that either exposes the patient to the possibility of potentially lethal GvHD or destroys the transplanted T cells before they can exert the desired effect [160]. To minimize the risk of immunological reactions to the cells, however, autologous CAR T cells are recommended; therefore, the currently accessible CAR T cell therapies are autologous, patient-specific varieties.

Additionally, there are further issues with CAR T therapy, including CRS, neurotoxicity, and off-target identification attacks [161,162,163]. Recent research indicates that up to 26% of CAR T cell-associated cardiotoxicities are believed to be largely driven by CRS. Cardiotoxicities manifest in a variety of ways but are associated with considerable morbidity and mortality and benefit from immediate immunosuppressive medication initiation [164]. A patient with relapsed diffuse large B cell lymphoma who relapsed rapidly after a previous autologous hematopoietic stem cell transplant has recently been diagnosed with progressive multifocal leukoencephalopathy months after lymphodepleting chemotherapy with fludarabine/cyclophosphamide and anti-CD19-directed CAR T therapy [165].

In the development of CAR-based treatments for autoimmune diseases, additional biological aspects must also be taken into account. Dysfunction of the immune system plays a role in the development of lymphoid tumors and autoimmune diseases, but in significantly different ways. Lymphomas are clonal diseases resulting from malignantly transformed cell proliferation, and we know the immunophenotype of the malignant cell clone. Hence, in these cases, it can be easier to target the characteristic molecular structure, thus, CAR construction could be slightly easier. However, in the vast majority of autoimmune diseases, the specific antigenic targets of autoantibodies are unknown. In certain instances, B cells produce autoantibodies against a nonpathogenic endogenous protein. The overlap in the autoantibody profile between many autoimmune diseases also complicates the understanding of the diseases. This makes it much more difficult to identify those structures (autoantigens) that can be targeted by CAR construction and whose targeting will lead to real clinical improvement in the respective diseases [166]. All these aspects make it difficult to develop CAR therapies suitable for autoimmune diseases. 

## 5. Conclusions

The management of autoimmune conditions and immune-mediated disorders as a whole is quickly changing, and novel strategies are currently being investigated. A better knowledge of pathogenic pathways and recent developments in cell production have led to the creation of new, specific treatments that significantly change interactions between cells and patient outcomes. Uncertain is the exact function of novel CAR-based treatments in the future therapeutic algorithm for autoimmune diseases.

Though we lack randomized data on the efficacy of CAR-based therapies in the treatment of autoimmune diseases, growing data from current studies already indicates that extensive immunosuppressive treatment could result in persistent results. These encouraging findings may pave the way for an additional ambitious therapy objective of obtaining extended remissions, which may become attainable in the near future. Similarly, findings from innovative CAR Treg-based therapies offering an immune modulation model are encouraging and might have a potential function in delivering clinical improvement with minimal toxicity. In the future, additional data will be needed for assessing the risk-benefit ratio for various CAR-based treatment options as well as to identify those who will profit most from such treatments.

## Figures and Tables

**Table 1:** The summary of the results of CAR-based treatments for autoimmune diseases and the key features and overall outcomes of ongoing and planned clinical trials.

Condition	Report or Trial Registry Number	Conditioning Treatment	CAR-Construct	Dosage of CAR Cells (Cells/kg Body Weight)	Overall Outcomes (or Primary Outcome Measures)	References
SLE (+ LN)	report	fludarabine	anti-CD19 CAR T	1.1 × 10^6^	- SELENA score decreased from 16 to 0 - dsDNA, C3, C4 levels normalized - patients achieved LLDAS - glucocorticoids were discontinued	[63,64]
SLE	report	fludarabine + cyclophosphamide	anti-CD19 CAR T	1 × 10^6^	- SLE remission according to DORIS criteria occurred within 3 months - SLEDAI median range became 0 - drug-free remission up to 8 months	[65]
SLE (+ stage IV DLBCL)	report	fludarabine + cyclophosphamide	anti-BCMA/CD19 compound CAR T (CD137 co-stimulation)	5.3 × 10^6^	- disease remission after 20 weeks - ANA was negative for 37 weeks	[66]
SLE	NCT03030976	cyclophosphamide	anti-CD19 CAR T (4-1BB co-stimulation)	10^6^–10^7^	- safety - efficacy	
SLE	NCT05030779	not available	anti-BCMA/CD19 compound CAR T	1–4 × 10^6^	- safety - efficacy	
SLE	NCT05474885	not available	anti-BCMA/CD19 compound CAR T	not available	- adverse effects	
SLE	NCT05765006	not available	anti-CD19 CAR T (Relma-cel)	15–150 × 10^6^	- safety - efficacy - pharmacokinetics - pharmacodynamics	
SS	NCT05085431	not available	anti-BCMA/CD19 compound CAR T	1–4 × 10^6^	- safety - efficacy - dose-limiting toxicity - adverse effects	
ANCA-associated vasculitis, AIHA (+ POEMS syndrome and amyloidosis)	NCT05263817	not available	anti-BCMA/CD19 compound CAR T	not available	- dose-limiting toxicity - safety - tolerability	
SSc	NCT05085444	not available	anti-BCMA/CD19 compound CAR T	not available	- dose-limiting toxicity - safety - tolerability	
MG	NCT04146051	not available	anti-BCMA CAR T (Descartes-08)	not available	- safety - tolerability - preliminary efficacy of a repeated dosing schedule of Descartes-08 CAR T	
MG, NMOSD, CIDP, IMNM	NCT04561557	fludarabine + cyclophosphamide	anti-BCMA CAR T (CT103A)	0.25–1 × 10^6^	- safety - dose-limiting toxicity - efficacy	
MG	NCT05451212	cyclophosphamide (+/− fludarabine)	anti-MuSK CAAR T	not available	- adverse events - dose-limiting toxicity	
PV	report		anti-DSG3 CAAR T	up to 2.5 × 10^9^	- no dose-limiting adverse effects - transient improvements in clinical disease activity and antibody levels with a duration of two months	[127]
PV	NCT04422912	intravenous immunoglobulin, cyclophosphamide (+/−fludarabine)	anti-DSG3 CAAR T	not available	- adverse events - dose-limiting toxicity	
CD, UC, DM, AOSD	NCT05239702	not available	anti-CD7 CAR T	not available	- dose-limiting toxicity - safety - tolerability	
IBD, GvHD (+ functional gastrointestinal disorders)	NCT03369353 (PREDICT trial)	not available	non-defined CAR T	not available	- applying a systems-biology approach to enable precision diagnostics for the key immunologic outcomes for the investigated disorders - investigation of the immunology of auto- and alloimmune gastrointestinal disorders - revealing the immune manifestations after CAR T therapy	

## Data Availability

No new data were created.

## Abbreviations

AChR: acetylcholine receptor; ACPA: anti-citrullinated protein antibodies; ACT: adoptive cell transfer; AIHA: autoimmune hemolytic anemia; alloCAR T: allogenic CAR T cell; ANA: antinuclear antibodies; AOM-DSS: azoxymethane-dextran sodium sulfate; AOSD: adult-onset Still’s disease; AP-1: activator protein 1 (Jun/Fos); APC: antigen-presenting cell; autoCAR T: autologous CAR T cell; BCMA: B cell maturation antigen; BCR: B cell receptor; ANCA: anti-neutrophil cytoplasmic antibody; Breg: regulatory B cell; BTK: Bruton tyrosin kinase; CAAR T: chimeric autoantibody receptor T cell; CAR NK: chimeric antigen receptor natural killer cell; CAR T: chimeric antigen receptor T cell; CAR Treg: chimeric antigen receptor regulatory T cell; Cas9: caspase 9; CASPR1: contactin-associated protein 1; cCAR T: compound CAR T cell; CD(number): cluster of differentiation; CD: Crohn’s disease; CEA: carcinoembryonic antigen; CIDP: chronic inflammatory demyelinating polyradiculoneuropathy; CNS: central nervous system; CNTN1: contactin 1; COVID-19: coronavirus disease 2019; CRISPR: Clustered Regularly Interspaced Short Palindromic Repeats; CRS: cytokine release syndrome; CTLA-4: cytotoxic T lymphocyte antigen 4; CV: citrullinated vimentin; DC: dendritic cell; DLBCL: diffuse large B cell lymphoma; DM: dermatomyositis; DNA: deoxyribonucleic acid; DORIS: definition of remission in SLE; Dsg: desmoglein; EAE: experimental autoimmune encephalomyelitis; FcR: fragment crystallizable receptor; FITC: fluorescein isothiocyanate; Foxp3: fork-box protein P3; GvHD: graft-versus-host disease; HIV: human immunodeficiency virus; HLA: human leukocyte antigen; HMGCR: 3-hydroxy-3-methylglutaryl-coA-reductase; HvGD: host-versus-graft disease; IBD: inflammatory bowel disease; IFN: interferon; Ig: immunoglobulin; IL: interleukin; IMNM: immune-mediated necrotizing myopathy; IPEX: immune dysregulation, polyendocrinopathy, enteropathy, X-linked syndrome; iPSC: induced pluripotent stem cell; ITAM: immunoreceptor tyrosine-based activation motif; JAK: janus-kinase; LLDAS: Lupus Low Disease Activity State; lpr: lupus-prone; LRP4: low-density lipoprotein receptor-related protein 4; MBP: myelin basic protein; MG: myasthenia gravis; MHC: major histocompatibility complex; MLV: murine leukemia virus; MOG: myelin oligodendrocyte glycoprotein; MRL: Murphy Roths Large; MS: multiple sclerosis; MuSK: muscle-specific tyrosine kinase; NF: neurofascin; NFAT: nuclear factor of activated T-cells; NKG2D: Natural killer group 2D; NMOSD: neuromyelitis optica spectrum disorder; NOD mice: non-obese diabetic mice; nTreg: natural Treg cell; NZBxNZW F1: New Zealand black x New Zealand white F1; PCreg: regulatory plasma cell; POEMS: Polyneuropathy, Organomegaly, Endocrinopathy, Monoclonal plasma cell disorder, Skin changes syndrome; pTreg: peripherally induced Treg cell; PV: pemphigus vulgaris; RA: rheumatoid arthritis; RF: rheumatoid factor; rh: recombinant human; RNA: ribonucleic acid; scFv: single chain fragment variant; SELENA: Safety of Estrogens in Lupus Erythematosus National Assessment; sIg: surface Ig; SLE: systemic lupus erythematosus; SLEDAI: SLE-disease activity score; SRP: signal recognition particle; SS: Sjögren’s syndrome; SSc: systemic sclerosis; STAT3: signal transducer and activator of transcription 3; T1D: Type 1 diabetes mellitus; TAA: tumor-associated antigen; Tc: cytotoxic T cell; TCR T: T cell receptor engineered T cell; TCR: T cell receptor; TGF: transforming growth factor; Th: helper T cell; TIL: tumor-infiltrating lymphocyte; TME: tumor microenvironment; TNBS: 2,4,6-trinitrobenzene-sulfonic acid; TNF: tumor necrosis factor; TNP: 2,4,6-trinitrophenyl; Treg: regulatory T cell; TRUCK: T cell Redirected for Antigen-Unrestricted Cytokine-initiated Killing; TYK2: tyrosine kinase 2; UC: ulcerative colitis; uniCAR T: switchable universal chimeric antigen receptor T cell; VIPER: Versatile ProtEase Regulatable.
